# Structural Defects, Mechanical Behaviors, and Properties of Two-Dimensional Materials

**DOI:** 10.3390/ma14051192

**Published:** 2021-03-03

**Authors:** Zixin Xiong, Lei Zhong, Haotian Wang, Xiaoyan Li

**Affiliations:** 1 Center for Advanced Mechanics and Materials, Applied Mechanics Laboratory, Department of Engineering Mechanics, Tsinghua University, Beijing 100084, China; xiongzx16@mails.tsinghua.edu.cn (Z.X.); zhongl15@tsinghua.org.cn (L.Z.); wanght14@mail.tsinghua.org.cn (H.W.); 2 Midea Group, Foshan 528311, China

**Keywords:** two-dimensional materials, mechanical behaviors, mechanical properties, structural defects, heterostructures, fracture

## Abstract

Since the success of monolayer graphene exfoliation, two-dimensional (2D) materials have been extensively studied due to their unique structures and unprecedented properties. Among these fascinating studies, the most predominant focus has been on their atomic structures, defects, and mechanical behaviors and properties, which serve as the basis for the practical applications of 2D materials. In this review, we first highlight the atomic structures of various 2D materials and the structural and energy features of some common defects. We then summarize the recent advances made in experimental, computational, and theoretical studies on the mechanical properties and behaviors of 2D materials. We mainly emphasized the underlying deformation and fracture mechanisms and the influences of various defects on mechanical behaviors and properties, which boost the emergence and development of topological design and defect engineering. We also further introduce the piezoelectric and flexoelectric behaviors of specific 2D materials to address the coupling between mechanical and electronic properties in 2D materials and the interactions between 2D crystals and substrates or between different 2D monolayers in heterostructures. Finally, we provide a perspective and outlook for future studies on the mechanical behaviors and properties of 2D materials.

## 1. Introduction

Two-dimensional (2D) materials are defined as crystalline materials consisting of single- or few-layer atoms, in which the in-plane interatomic interactions are much stronger than those along the stacking direction. Since the first exfoliation of single-layer graphene [1], 2D materials have attracted worldwide attention due to their unique structures and remarkable properties [2,3,4,5,6,7]. For example, graphene composed of hexagonally arranged sp^2^ hybridized atoms possesses extraordinary strength [8], giant carrier mobility [9], extremely high thermal conductivity [10], and excellent optical properties [11,12] compared to the existing materials. These exceptional properties and single-atomic-layer structures enable graphene to have a wide range of applications in field-effect transistors [13,14,15], flexible electronics [16,17], photodetectors [18,19,20,21], composite materials [22], energy storage [23,24,25], precise sensors [26,27,28], DNA sequencing [29,30,31] and drug delivery [32,33,34].

The rapid and prosperous development of graphene stimulates numerous research interests on other 2D materials. More than one thousand structures of 2D materials have been predicted to be easily exfoliated to monolayers or multilayers with fascinating physical properties, forming a large family of 2D materials [35]. The booming synthetic methods established from graphene have brought experimental realizations of dozens of novel 2D crystals. Monolayer MoS_2_ [36] and hexagonal boron nitride (h-BN) [37,38] have been extracted at an early stage and have recently received much attention. Some graphene analogs such as black phosphorene [39], borophene [40,41], silicene [42,43], germanane [44], stanene [45], antimonene [46], bismuthene [47,48] and tellurene [49] have been synthesized in the past few years. Although these 2D materials have an atomic layer structure similar to that of graphene, their physical properties are distinct from those of graphene. Thus, these 2D materials can act as complementary materials and have the potential for broader applications. For example, unlike graphene, phosphorene has a strong in-plane structural anisotropy, leading to a significant dependence of the material properties on its orientation [39,50]. For electronic properties, graphene has a direct zero band gap and exhibits a certain metallicity. Other 2D crystals have a large variety of band structures. The direct band gaps of h-BN [51], MoS_2_ [52,53,54], and WSe_2_ [55] allow them to be promising materials for optical devices, transistors, phototransistors, and photodetectors. The metallic electronic character possessed by borophene [56,57] and VS_2_ [58] is essential for electronic and energy storage applications. In addition, stanene, as a 2D topological insulator, is theoretically predicted to display superconductivity at the edges [59]. A large number of 2D material family members could satisfy variant requirements for a huge diversity of applications. The structure and mechanics of 2D materials play important roles in manufacturing, integration, and performance for their potential applications. In this paper, we review the recent advances in the intrinsic microstructures and unique mechanics of 2D materials (including graphene and other 2D crystals) to provide a fundamental understanding of their mechanical behaviors and properties.

During the synthesis of 2D materials, various types of defects are inevitably generated. For example, during the chemical vapor deposition (CVD) process for the large-area growth of graphene, many isolated grains from different nucleation sites stitch into uniform structures, leading to the formation of grain boundaries (GBs) between neighboring grains with a misorientation [60,61]. Furthermore, graphene’s irradiation or chemical treatment can generate various point defects, such as dislocations, vacancies, and functionalized groups [62,63,64]. The majority of experimental studies have shown that these defects in 2D crystals significantly affect their physical, chemical, and mechanical properties [65,66]. In particular, it has been demonstrated that defects can tailor the properties of 2D materials via the controlled arrangement of defects [65,66]. Therefore, the concepts of defect engineering and topological design have emerged and been used to achieve tunable properties of 2D materials.

In this review, we first summarize the atomic structures of numerous 2D materials and provide a general classification based on their atomic structures in Section 2. Then, we highlight some common defects in several representative 2D crystals in Section 3. In Section 4, we introduce recent experimental, computational, and theoretical studies on the mechanical properties and behaviors of 2D materials, emphasizing deformation and fracture mechanisms and the influence of various defects on mechanical behaviors and properties. We also discuss the piezoelectric and flexoelectric behaviors of specific 2D materials and the interactions between 2D crystals and substrates or between different 2D monolayers. In the final section, we provide a perspective and outlook for future studies on the mechanical behaviors and properties of 2D materials.

## 2. Classification and Atomic Structures

**Figure 1:**
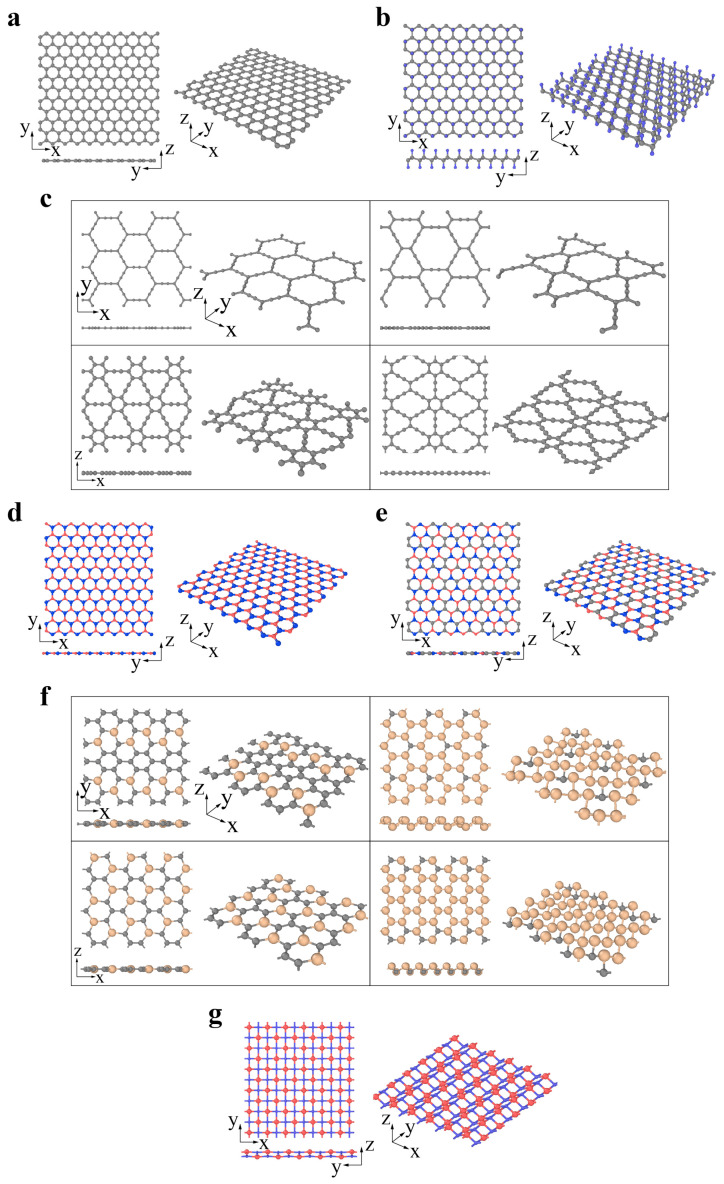
The graphene family: (**a**) graphene (gray atom represents C), (**b**) CX (X = H, F, Cl; gray and blue atoms represent C and X, respectively), (**c**) graphyne (α-graphyne, β-graphyne, γ-graphyne and 6,6,12-graphyne from left to right, top to bottom; gray atom represents C), (**d**) h-BN (red and blue atoms represent B and N, respectively), (**e**) BCN (reproduced from Ref. [71]; red, gray and blue atoms represent B, C and N, respectively), (**f**) Si*_x_*C*_1−x_*(*x* = 2/10, 5/6, 2/6, 14/18 from left to right, top to bottom; gray and yellow atoms represent C and Si, respectively) (reproduced from Ref. [72]), (**g**) TiC (reproduced from Ref. [73]; red and blue atoms represent C and Ti, respectively).

The 2D material family has extended to more than one thousand members based on theoretical predictions [35]. To date, tens of these materials have been synthesized experimentally [35]. Generally, 2D materials can be categorized into four types (including graphene family, Xenes, chalcogenides, and 2D oxides) according to their components and atomic structures, as shown in Figure 1.

The graphene family contains graphene and its derivatives consisting of different hybridized carbon atoms or heterogeneous elements, as illustrated by Figure 1a–g. In fluoro-graphene, chloro-graphene, and graphene oxide, the saturated carbon atoms (sp^3^ hybridization) bind with noncarbon elements, forming an alternating pattern. Carbon allotropes (such as graphyne) are constructed by the network of sp- and sp^2^-hybridized carbon atoms. The graphyne structure can be regarded as replacing partial aromatic C–C bonds in graphene with acetylene chains. Complete, 2/3, 1/3 and 5/12 replacements result in α-, β-, γ- and 6,6,12-graphyne, respectively (Figure 1c) [67,68]. Graphyne structures exhibit fascinating semiconducting properties, enabling their use in electronic devices [69,70]. These structures are also thought to be possible candidates in gas separation, filtration, and water desalination because of their intrinsic nanopores [69,70]. Analogous to graphene, two or more elements can substitute the original carbon atoms to form more complex layered systems, such as h-BN (Figure 1d), boron–carbon–nitrogen (BCN) (Figure 1e) [71], and Si*_x_*C*_1−x_* (Figure 1f) [72]. In addition to the analogous hexagonal structure above, a 2D material with a tetragonal arrangement is also predicted to be considerably stable. The p(pi)–d(pi) bonded TiC (Figure 1g) is buckled into a zigzag line in the side view. Such a distinguished structure endows it with anisotropic properties [73].

**Figure 2:**
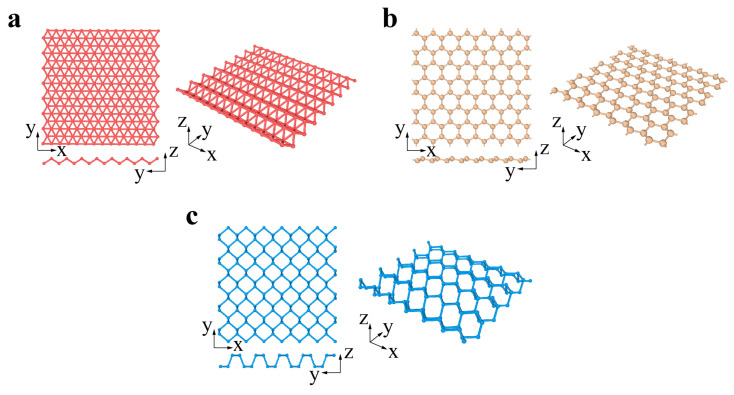
Xenes: (**a**) borophene, (**b**) silicene, germanene, stanene and antimonene, (**c**) phosphorene.

Xenes are monoelement 2D materials organized into distorted hexagonal or trigonal lattices. The 2D Xenes can be made up of group IIIA, IVA, and VA elements, termed borophene, silicene, germanene, stanene, phosphorene, and antimonene when X = B, Si, Ge, Sn, P, and Sb, respectively (Figure 2a–c). Unlike the ideally flat structure of graphene, 2D Xenes prefer alternating out-of-plane atomic arrangements, resulting in an anisotropic lattice structure. Due to abundant components and unique structures, 2D Xenes exhibit excellent physical, chemical, and mechanical properties, enabling them to be promising agents for biosensors, bioimaging, therapeutic delivery, and theranostics [74].

**Figure 3:**
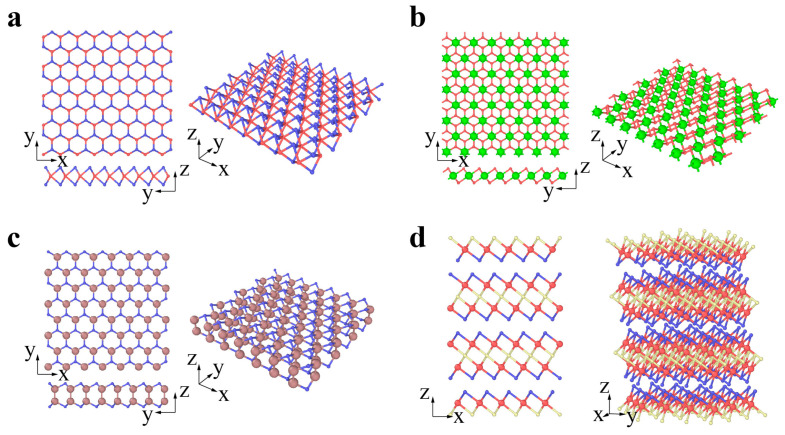
Chalcogenides: (**a**) 2H-MX_2_ (M = Mo, W, Nb, Ta; X = S, Se, Te; red and blue atoms represent M and X, respectively), (**b**) 1T-MX_2_ (M = Zr, Hf; X = S, Se; green and red atoms represent M and X, respectively), (**c**) GaS, GaSe and InSe (brown and blue atoms represent Ga or In and S or Se, respectively), (**d**) Bi_2_Se_3_, Bi_2_Te_3_ and Sb_2_Te_3_ (red atoms represents Bi or Sb, while blue and yellow atoms represent Se or Te in different layers, respectively).

Chalcogenides are a type of emerging 2D material represented by the transitional metal dichalcogenide (TMDC) MX_2_. For MX_2_, the layer of transitional metal atom M (Mo, W, Nb, Ta) is sandwiched by two layers of chalcogen atoms X (S, Se, Te). MX_2_ usually has two typical phases: 2H and 1T phases [75,76,77]. The 2H phases have been widely studied to date. The MX_2_ (M = Mo, W, Nb, Ta; X = S, Se, Te) in Figure 3a prefers to be the 2H phase in equilibrium, while the MX_2_ (M = Zr, Hf; X = S, Se) in Figure 3b prefers to be the 1T phase [78,79]. The transformation from 2H to 1T phases can occur under specific conditions [75,76,77]. GaS, GaSe, and InSe are chalcogenides with a double layer of metal intercalated between two layers of chalcogen, forming an X-M-M-X vertical structure (Figure 3c) [80]. Bi_2_Te_3_, Bi_2_Se_3_, and Sb_2_Te_3_ belong to a specific branch of chalcogenides and are called topological insulators. There exists a van der Waals interaction between the stoichiometric monolayers, e.g., quintuple layers (QLs). A Bi_2_Se_3_ QL comprises five atomic layers stacked in the sequence of Se (1)-Bi-Se (2)-Bi-Se (1) along the c-axis (Figure 3d) [81].

**Figure 4:**
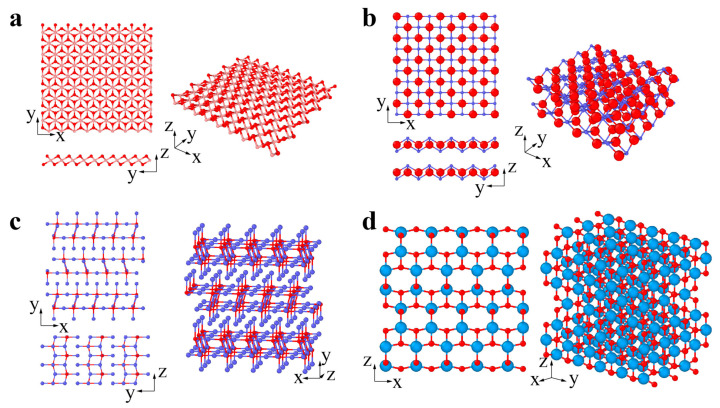
2D oxides: (**a**) MnO_2_ (pink and red atoms represent Mn and O, respectively), (**b**) PbO (blue and red atoms represent Pb and O, respectively), (**c**) MoO_3_ (red and blue atoms represent Mo and O, respectively), (**d**) TiO_2_ (azure and red atoms represent Ti and O, respectively).

The common types of 2D oxides include lead, phosphorus, and transition metal oxides (Figure 4a–d) [82,83]. 2D oxides usually appear as single planar structures and multilayer and superlattice structures (Figure 4a–d). Layered 2D oxides have strong lateral chemical bonding in planes but exhibit weak van der Waals interactions between layers, while nonlayered 2D oxides (with superlattice structures) have atomic bonding in three dimensions [84]. Many 2D oxides are functional materials with great potential in catalysis, energy storage, and electronics, since they have a highly chemically active interface [84].

## 3. Structural Defects in Various 2D Materials

The imperfections introduced during production processes inevitably distort the pristine lattices and have a significant influence on the mechanical properties of materials. The accurate manipulation of defects has been recognized as an effective approach to alter/modify the mechanical properties of materials. Structural defects in 2D materials have attracted much attention since the era of graphene has arisen. In particular, design based on topological defects in 2D materials has become a hot topic in the field of mechanics and material sciences [85,86]. However, extensive studies have mainly focused on defective graphene [85,86], and there have been few studies on the defects of newly synthesized 2D materials. Due to the confinement from the reduced dimensionality, topological defects in 2D materials are less abundant and complicated than those in bulk structures. Similar to the defect types in bulk materials, the defects in 2D structures can be classified as point defects, dislocations, and GBs. In this section, we address the morphology and energetic features of some typical defects in several representative 2D materials.

### 3.1. Defects in Graphene and h-BN

#### 3.1.1. Point Defects

**Figure 5:**
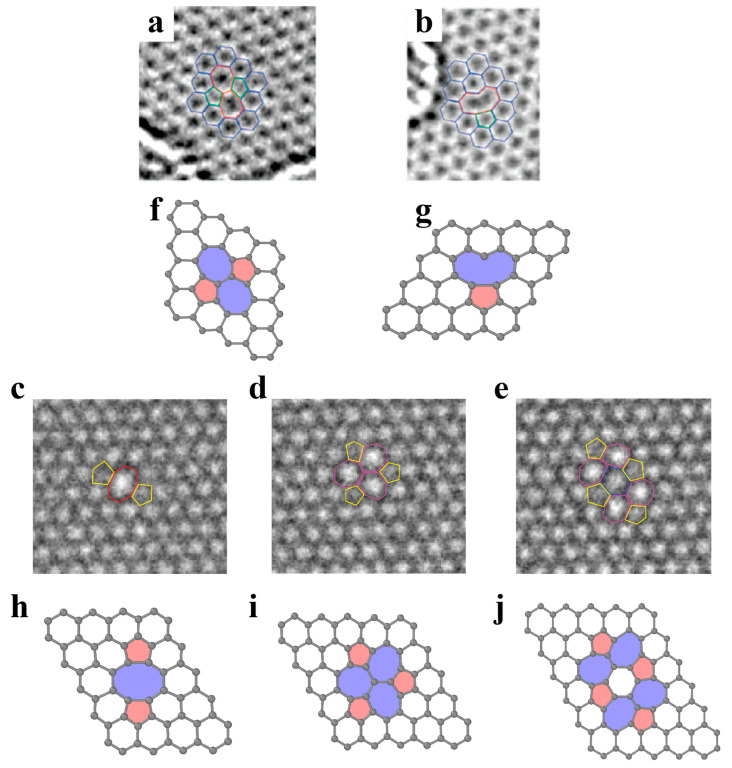
Point defects in graphene. (**a**) TEM image of a Stone–Wales defect, reproduced from Ref. [90]. (**b**) TEM image of a single vacancy reproduced from Ref. [90]. (**c**–**e**) TEM image of double vacancies reproduced from Ref. [94]. (**c**) V_2_ (5-8-5) defect, (**d**) V_2_ (555-777) defect, (**e**) V_2_ (5555-6-7777) defect. (**f**–**j**) Atomic structures of defects corresponding to images (**a**–**e**).

**Figure 6:**
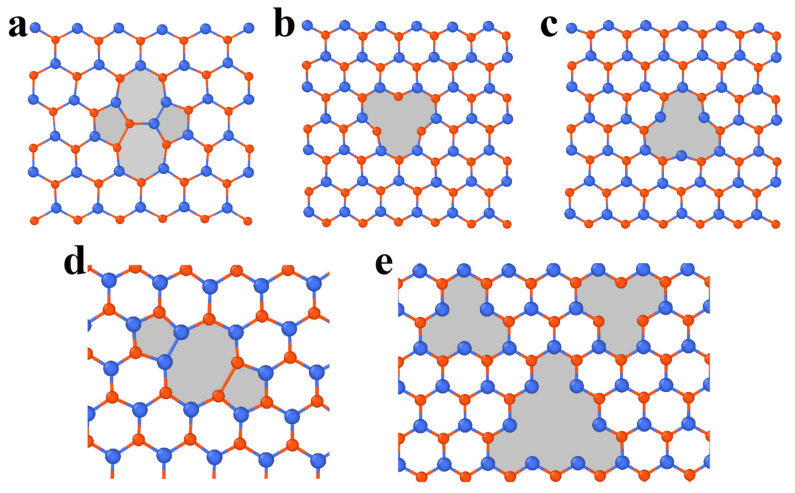
Point defects in h-BN. (**a**) Atomistic structure of a Stone–Wales defect. (**b**–**e**) Atomistic structure of vacancies. (**b**) Single vacancy V_N_. (**c**) Single vacancy V_B_. (**d**) Double vacancy V_BN_. (**e**) Vacancy of V_B_, V_N_ and V_3B+N_. Red and blue atoms represent B and N, respectively.

As shown in Figure 5a,f, the Stone–Wales defect involves the 90° rotation of a pair of atoms in graphene and results in the formation of two pentagons and two heptagons, which are regarded as two back-to-back dislocation cores. The generation of such defects does not involve the addition or loss of atoms, and no dangling bonds are introduced. The formation energy of Stone–Wales defects in graphene is approximately 5 eV [87,88]. Such high formation energy implies a nonequilibrium condition for the observation of Stone–Wales defects. The h-BN structure presents the same planar hexagonal lattice with alternatively positioned boron and nitrogen atoms. The Stone–Wales defects in the h-BN structure (Figure 6a) are less energetically favorable since they involve two sets of new homoelemental bonds. The formation energy of Stone–Wale defects in h-BN reaches up to 7.28 eV [89].

A single vacancy in graphene induces Jahn–Teller distortion and the formation of a five-membered ring, a nine-membered ring (V_1_ (5-9) defect), and one dangling bond [90] (Figure 5b,g). The formation energy of a single vacancy is up to 7.6 eV, while its migration barrier is approximately 1.3 eV [91,92,93]. Divacancy leads to a two pentagons and one octagon [V_2_ (5-8-5) defect] arrangement, with a formation energy of 8.7 eV [87]. The double vacancy can further transform into a combination of three pentagons and three heptagons (V_2_ (555-777) defect) or four pentagons and four heptagons (V_2_ (5555-6-7777) defect) (Figure 5c–e,h–j), which has been captured experimentally [94]. The formation energy of 555-777 defects is lower than that of 5-8-5 defects [95], while the formation energy of 5555-6-7777 defects is between those of 5-8-5 and 555-777 defects [96]. The divacancy migration energy is approximately 7 eV [92]. Therefore, the double vacancy is more stable and less movable than the single vacancy in graphene.

The vacancy in h-BN contains boron (V_B_) or nitrogen (V_N_) single vacancies and double vacancies (V_BN_) (Figure 6b–d). The formation energies of V_B_, V_N_ and V_BN_ are approximately 10.0 eV, 8.3 eV and 11.73 eV (5-8-5), respectively [97,98,99]. The equilibrium configurations of a single vacancy have three-fold symmetry. The V_BN_ has relatively low formation energy among vacancy pairs, suggesting that it is the energetically preferred and experimentally observed defect [97,99]. Compared with graphene, the divacancies in h-BN are less favorable. Tetravacancy V_3B+N_ is also observed in monolayer h-BN (Figure 6e), which might be transformed from V_BN_ by further removing boron atoms under irradiation [100].

#### 3.1.2. Dislocations and Grain Boundaries

**Figure 7:**
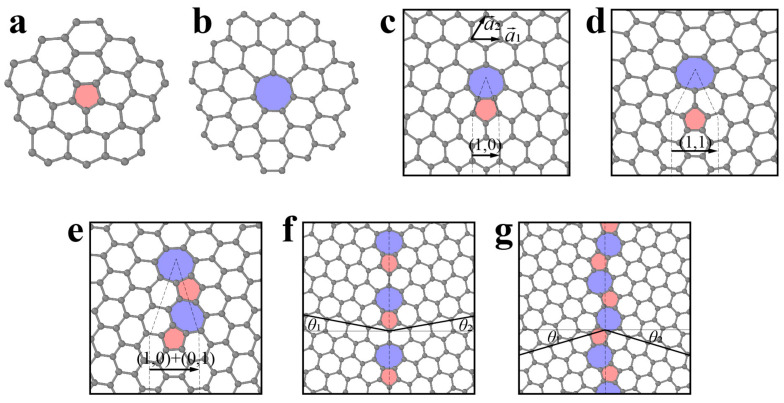
(**a**,**b**) Atomistic structure of disclinations in graphene. (**c**,**e**) Atomistic structure of dislocations in graphene. (**c**) (1,0) dislocation, (**d**) (1,1) dislocation, (**e**) (1,0) + (0,1) dislocation. (**f**,**g**) Atomistic structure of grain boundaries in graphene.

**Figure 8:**
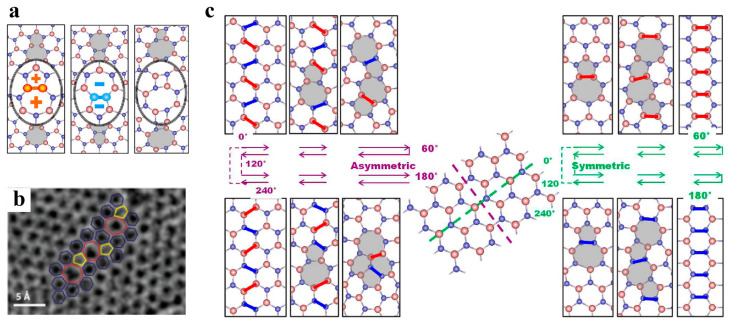
(**a**) Atomistic structure of the dislocation pair in h-BN, reproduced from Ref. [104]. (**b**) high-resolution transmission electron microscopy image of the grain boundary, reproduced from Ref. [106]. (**c**) Atomic structure of grain boundaries with various tilt angles, reproduced from Ref. [104]. Red and blue atoms represent B and N, respectively.

When a semi-infinite 60° wedge of material is removed from or inserted into pristine graphene, disclination with an isolated pentagon or heptagon carbon ring is generated (Figure 7a,b) [101,102]. The introduction of a single disclination leads to substantial global deformation and therefore is energetically unfavorable. Analogous to bulk crystals, dislocations are defined in 2D materials. A dislocation is considered as inserting semi-infinite strips into the original structure and is equivalent to a pair of positive and negative disclinations (i.e., dislocation cores). The Burges vector of dislocations in graphene can be expressed as a translation vector of the atomic lattice, whose magnitude represents the width of the embedded strip (Figure 7c–e). As illustrated in Figure 7c–e, the typical Burger vectors of dislocations in graphene include (1,0), (1,1) and (1,0) + (0,1). Due to the limitation of two dimensions, there is only edge dislocation in 2D materials [103]. In graphene, the common dislocation is a pair of pentagon–heptagons (5|7), which has been observed experimentally [61]. Recent first-principle calculations showed that for h-BN, square–octagon (4|8) dislocations are more stable than common pentagon–heptagon (5|7) dislocations due to avoidance of unfavorable homoelemental bonding [104], as shown in Figure 8a. However, the 4|8 dislocation induces the buckling of the free-standing layer in the out-of-plane direction, which effectively relaxes the strain from the dislocation core [104].

In three-dimensional (3D) crystalline materials, GB is a common planar defect and separates two gains with different orientations. The misorientation is an important parameter to characterize GB and is usually denoted by an angle. In the 2D lattice, the GB is a line array of dislocation cores, as illustrated in Figure 7f,g and Figure 8b. The structures of energetically favorable GBs can be determined by first-principle calculations and further confirmed by high-resolution transmission electron microscopy (HR-TEM) observations [61,103,105]. The GB energy per length significantly depends on the misorientation (i.e., tilt angle *θ* between two grains). When the tilt angle is smaller than 10° (i.e., in the low-angle regime), the GB energy generally increases with increasing tilt angle, following the Read–Shockley equation [103]. In the large-angle regime, GB energy at specific angles can be reduced significantly by out-of-plane buckling due to the presence of dislocations [103]. Due to the heterogonous elements in the atomic structure, the GBs in monolayer h-BN can be classified into two groups: asymmetric GBs (asym-GBs) and GBs with mirror symmetry (sym-GBs) (Figure 8c). The former is composed of 4|8 dislocation pairs, while the latter contains 5|7 pairs, and has been validated via HR-TEM [104,106]. Sym-GBs with elemental polarity carry net charges due to the ionic nature of boron–nitrogen bonds, which may endow new applications in electronics [104].

### 3.2. Defects in MoS_2_

#### 3.2.1. Point Defects

**Figure 9:**
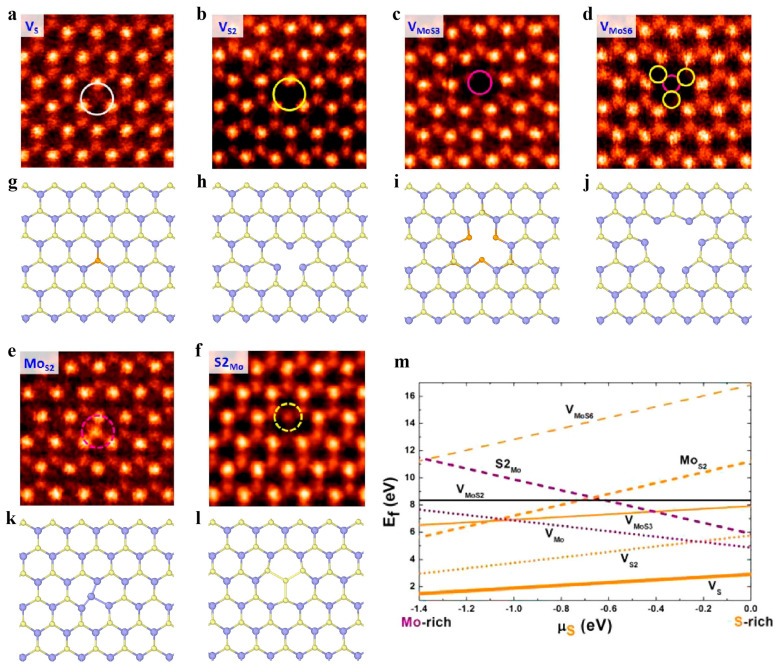
Point defects in MoS_2_. Atomic-resolution annular dark field (ADF) images of (**a**) monovacancy of sulfur (V_S_), (**b**) divacancy of sulfur pairs (V_S2_), (**c**) vacancy complex composed of single Mo with related three sulfur (V_MoS3_), (**d**) Mo with nearby three disulfur pairs (V_MOS6_), (**e**) Mo_S2_, and (**f**) S2_Mo_ reproduced from Ref. [107]. (**g**–**l**) Optimized atomic structures from density functional theory (DFT) calculations of defects corresponding to images (**a**–**f**), reproduced from Ref. [107]. (**m**) Formation energy of defects with different sulfur chemical potentials, reproduced from Ref. [107]. Purple, yellow and orange atoms represent Mo, top layer S and bottom layer S, respectively.

MoS_2_ is one of the most representative materials among various TMDCs with sandwiched atomic arrangements. The common point defects in the MoS_2_ structure contain four types of vacancies and two types of antisite defects. The vacancy defects include monovacancy of sulfur (V_S_), divacancy of sulfur pairs (V_S2_), and vacancy complex composed of single Mo with related three sulfur (V_MoS3_) or Mo with nearby three disulfur pairs (V_MoS6_). (Figure 9a–d,g–j). The antisite defects can be termed as Mo_S2_ (i.e., a Mo atom substituting an S_2_ column) or S_2_Mo (i.e., a Mo atom being replaced with an S_2_ column) (Figure 9e,f,k,l) [107]. V_S_ is found by first-principle calculations to have the lowest formation energy regardless of S chemical potential, while Mo_S2_ and S_2_Mo antisite defects present almost the highest formation energies in a wide range of chemical potentials (Figure 9m). The energy analyses from the calculation indicate that Vs is easily observed, while antisite defects rarely occur [107], which is consistent with the experimental observations.

#### 3.2.2. Dislocations and Grain Boundaries

**Figure 10:**
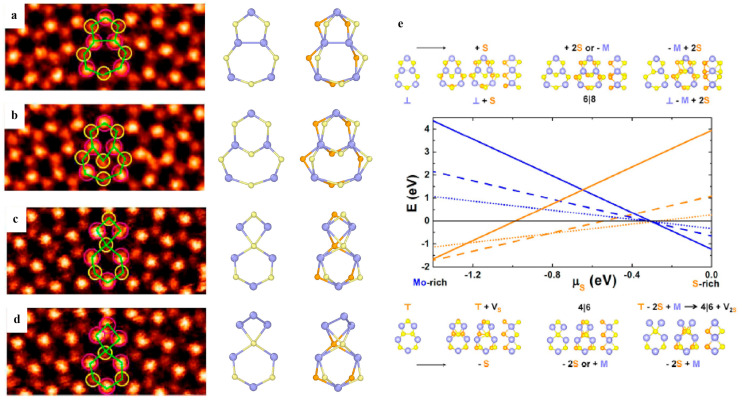
Dislocations in MoS_2_. ADF image and atomic structure of (**a**) 5|7 dislocation, (**b**) 6|8 dislocation, (**c**) pristine 4|6 dislocation, and (**d**) 4|6 with Mo substitution, reproduced from Ref. [107]. (**e**) Energies of different dislocations as functions of sulfur chemical potential, reproduced from Ref. [108]. Purple, yellow and orange atoms represent Mo, top layer S and bottom layer S, respectively.

The sandwiched atomic arrangements complicate the dislocation structures in MoS_2_. Calculations based on density functional theory (DFT) showed that the dislocation cores in MoS_2_ have concave three-dimensional (3D) polyhedra of polyelemental composition and generally exhibit dreidel-like shapes [108]. The 5|7 dislocation core is a typical dislocation structure in 2D materials composed of hexagonal rings. Due to elemental heterogeneity, the 5|7 pairs in MoS_2_ have both Mo-rich and S-rich types with homoelemental bonds. A 4|8 dislocation in MoS_2_ has a larger Burgers vector than the common 5|7 dislocation, consequently with higher elastic deformation energy [108]. The first principle calculations revealed that an isolated 4|8 dislocation is unstable and decomposes into two 5|7 dislocations, where one has an M-rich core and the other has an S-rich core to preserve the elemental balance [108]. The derivative dislocation cores (including 4|6 and 6|8) in MoS_2_ can be obtained by the insertion, substitution, or deletion of the specific atoms in the 5|7 dislocations (Figure 10a–d). The formation energies of these derivative defects are related to the chemical equilibrium conditions, as shown in Figure 10e [108]. Direct atomic resolution imaging has confirmed various dislocation core structures in MoS_2_ and other TMDCs [107,109,110].

**Figure 11:**
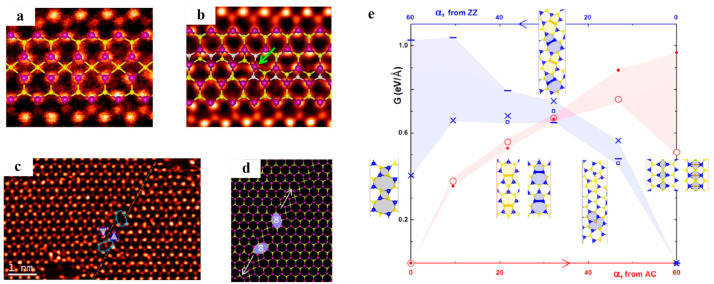
Grain boundaries in MoS_2_. ADF image of (**a**) 4|4P grain boundary (GB), (**b**) 4|4E GB and (**c**) 4|4P GB kinks, reproduced from Ref. [107]. (**d**) Atomic structure of 4|4P GB kinks corresponding to image (**c**), reproduced from Ref. [107]. (**e**) Variation in GB energy with tilt angle, reproduced from Ref. [108].

Similar to h-BN, there are also two distinct GB structures, including asymmetric GBs (asym-GBs) and symmetric GBs (sym-GBs), in MoS_2_, but their atomic configurations are more complex than those in h-BN. HR-TEM observations showed that a sym-GB with a misorientation of 60° in MoS_2_ is composed of four-fold rings sharing a point at a common S2 site (denoted as 4|4P) (Figure 11a) [107]. Previous DFT calculations predicted that such GBs could serve as one-dimensional metallic stripes embedded in semiconducting MoS_2_, which form intrinsic electronic heterostructures and further endow new functionalities [108]. The GBs in polycrystalline MoS_2_ usually exhibit a wavy path instead of a perfectly straight line. Therefore, the octagons are observed to connect the parallel segments of 4|4P GBs to achieve GB kinks (Figure 11c,d) [107,111]. Another type of sym-GB structure is composed of four-fold rings sharing an edge (denoted as 4|4E) (Figure 11b) and has very close energy to the 4|4P structure [107]. Different from 4|4P GBs, two 4|4E GBs are linked by a four-fold coordinated Mo to form GB kinks [107]. The asym-GBs in MoS_2_ have a more complex geometry than sym-GBs. It is noted that the asym-GBs have no polarity because they have equal amounts of Mo and S [107]. However, sym-GBs are polar because they have an excess of one of the elements [107]. As shown in Figure 11e, the GB energy per unit length in MoS_2_ linearly increases with increasing tilt angle in the initial regime. However, at a large tilt angle, the GB energy exhibits a nonlinear dependence on the tilt angle and even decreases, which is attributed to the complex interaction between dislocations in the GB [108].

### 3.3. Defects in Black Phosphorus and Borophene

#### 3.3.1. Point Defects

**Figure 12:**
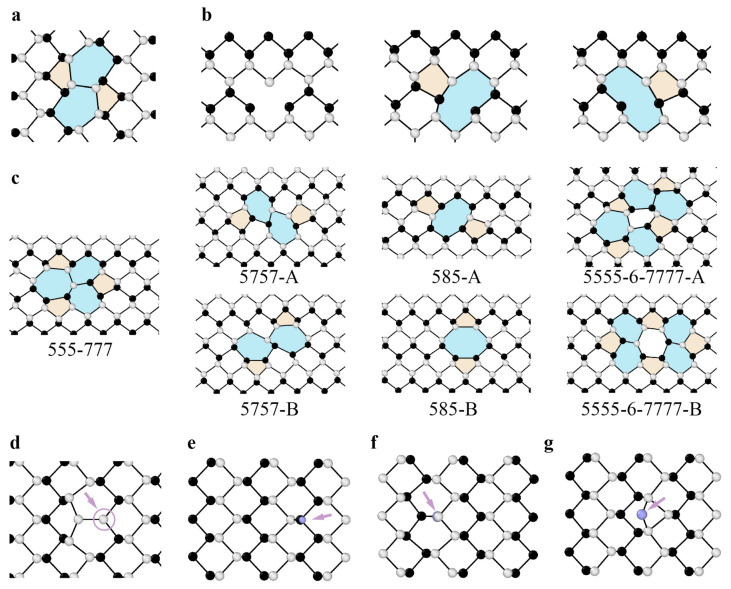
Point defects in phosphorene. (**a**) Stone–Wales defect. (**b**) Various states of single vacancy. (**c**) Various states of double vacancies reproduced from Ref. [115]. (**d**) Self-interstitial. (**e**) Dangling oxygen. (**f**) Interstitial oxygen. (**g**) Horizontal oxygen bridge. (**e**–**g**) Reproduced from Ref. [118]. Black and write atoms represent P in different layers, while purple atom represents O.

Noncarbon 2D materials with a single element have also attracted tremendous attention due to their unique structures and distinct properties. The phosphorus monolayer exfoliated recently exhibited fascinating electronic properties for future applications [39,112,113,114]. Unlike the aforementioned materials with hexagonal symmetry, phosphorene has rectangular symmetry with buckled hexagonal atomic arrangements. In phosphorene, point defects (such as Stone–Wales defects, vacancies, and self-interstitial defects) have been investigated by DFT calculations (Figure 12a–g) [115,116]. The Stone–Wales defects induced by bond rotation lead to two puckering pentagon and heptagon pairs (Figure 12a). Similar to graphene, the single vacancy in phosphorus results in 5-9 defects driven by Jahn–Teller distortion (Figure 12b). The formation energy of monovacancies is up to 1.65 eV, which is much lower than that (7.57 eV) of graphene [115,116]. The divacancy in graphene can transform from 5-8-5 defects to 555-777 defects or 5555-6-7777 defects, which also take place in phosphorene monolayers. Due to the puckering structure, the 5-8-5 and 5555-6-7777 defects further evolve into two topologically equivalent conformers named types A and B (Figure 12c) with considerably large differences in formation energy [115]. Interestingly, the formation energy of divacancies is comparable to or even lower than that of monovacancies [115,116,117], indicating an energetically spontaneous coalescence of monovacancies. Compared with the energy barrier in graphene, h-BN, and MoS_2_ mentioned above, the formation energies of vacancies in phosphorene are much lower, which is attributed to the weaker P–P bond and the puckered structure [115].

Interstitial defects usually appear during the growth of 2D phosphorus sheets. The self-interstitial P atom prefers to form two bonds with P atoms in different layers, leaving one dangling bond [116]. As a result, the formation energy of self-interstitial phosphorus is much lower than that of graphene (Figure 12d) [116]. To investigate the stability in air exposure, the absorption of O atoms in phosphorus monolayers has been studied by first-principle calculations [118]. The O atom can form various structures (including dangling and interstitial oxygen and horizontal oxygen bridges) with pristine lattices, leading to minor distortions of the lattice. The absorption process of O atoms in dangling or interstitial positions is exothermic, while the process for horizontal bridge positions is metastable (Figure 12e–g) [118]. Note that the O atoms exist in terms of molecules in the atmosphere; hence, the O_2_ molecule first dissociates and then reacts with P atoms in phosphorene [118,119]. First-principle calculations revealed that the surface reaction with oxygen could lead to the degradation of phosphorene exposed to ambient conditions. Therefore, when phosphorene is used in practical applications, it cannot be exposed to ambient conditions and requires encapsulation.

**Figure 13:**
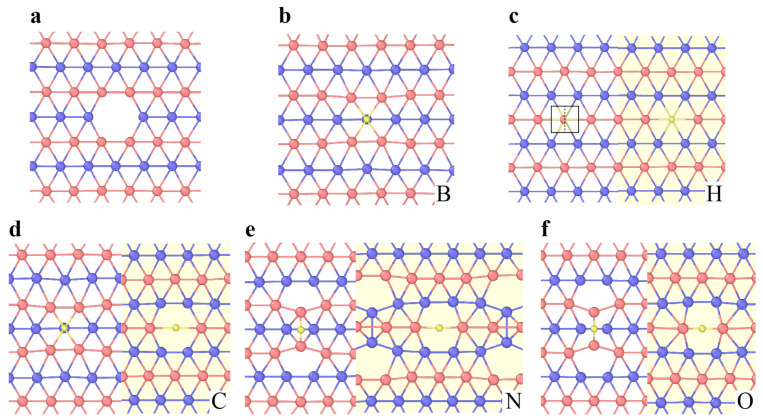
Point defects in borophene. (**a**) Monovacancy. (**b**–**f**) Interstitial and substitutional (shadowed as yellow) defects of B, H, C, N, and O, reproduced from Ref. [120]. Red and blue atoms represent B in top and in bottom layers.

Although there are many allotropes for boron, the boron monolayer on the silver surface under ultrahigh vacuum conditions was successfully synthesized in 2015 [41]. Boropene has an undulating triangular grid and exhibits highly anisotropic properties. The monovacancy in borophene (Figure 13a) has formation energy as low as 0.1 eV, indicating that it is one of the most common defect structures. The uniform distribution of vacancies in borophene has been confirmed via first-principle calculations due to the Coulomb interactions caused by vacancies [120]. The influences of substitution and insertion of H, C, N, and O (Figure 13b–f) have been recently investigated through DFT calculations combined with a semiempirical van der Waals dispersion correction [120]. The lattice distortions and charge density redistribution of these substitutional and interstitial point defects significantly depend on the element types and the temperature. The energy barriers of O and N doping processes are lower than 0.3 eV, suggesting the reactivity of 2D boron sheets with air [120].

**Figure 14:**
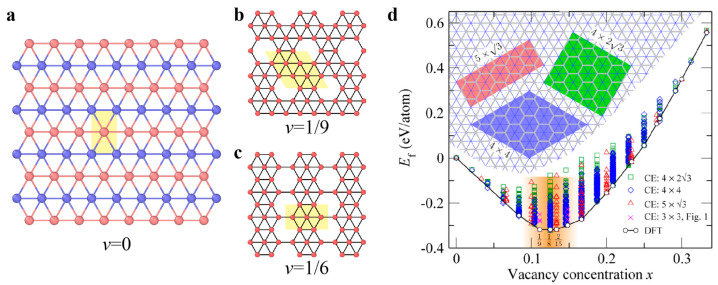
(**a**–**c**) Polymorphs structures with variant fractions in borophene. (**d**) Formation energy of various borophene phases, reproduced from Ref. [121]. Red and blue atoms represent B in top and in bottom layers in (**a**), and red atom represents B in (**b**) and (**c**).

**Figure 15:**
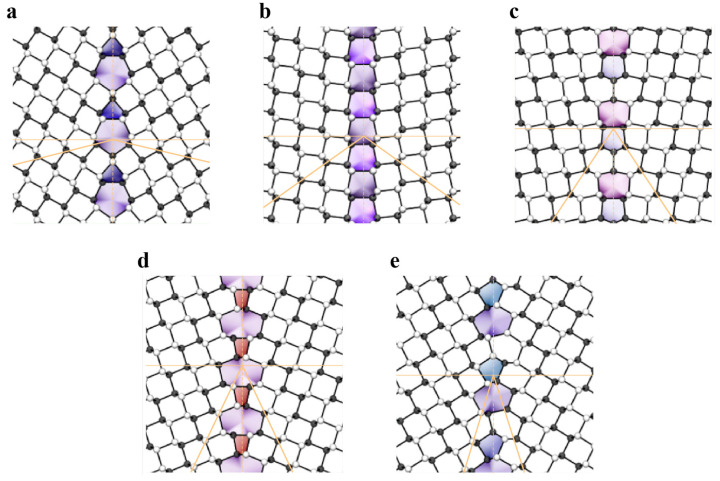
Grain boundaries in phosphorene. (**a**–**e**) Atomic structure of GB with various tilt angles. Black and write atoms represent P in different layers.

Unlike other 2D crystals, multiple polymorphs of borophene with different arrangements of periodic hexagonal holes (where hole density is characterized by the parameter *v*) have been recently discovered from both theoretical predictions [121] and experimental observations [40] (Figure 14a–c). The structures of these polymorphs are dependent on the synthesis conditions and substrates [122,123,124,125]. Among a large number of possible borophene phases, the *v*_1/9_ structure is predicted to be the most stable [121,126,127]. First-principle calculations have revealed low formation energy of single vacancies in various borophene polymorphs, as shown in Figure 15d. The reactivity of doping and absorbing different elements in borophene polymorphs is mainly sensitive to their structure. The difference in the energy barrier for different substitutional doping and adatom adsorption is attributed to distinct chemical environments of B atoms [127].

#### 3.3.2. Dislocations and Grain Boundaries

**Figure 16:**
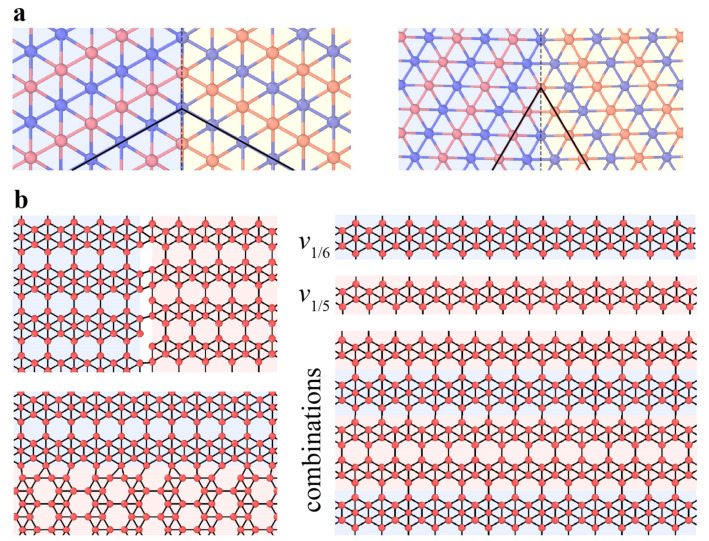
(**a**) Atomic structure of tilt GBs in borophene. Red and blue atoms represent B in top and in bottom layers. (**b**) Formation of new borophene phases, reproduced from Ref. [129]. Red atom represents B.

Similar to the hexagonal 2D materials, the primary dislocation in phosphorene is 5|7 pairs with a buckled structure (Figure 15). The GB in phosphorene contains an array of dislocations, and the tilt angle determines the GB energy. The GBs in phosphorene are more stable than those in graphene due to the much lower formation energies of dislocations constituting GBs [116,128]. The line defects (i.e., *v*_1/6_ and *v*_1/5_ rows) found in borophene structures have similar lattices and can serve as the building blocks of a number of borophene crystalline phases by controlling the mixing ratio of the two rows (Figure 16) [129]. The registry of the lattices along the horizontal directions of rows *v*_1/6_ and *v*_1/5_ allows the *v*_1/6_ and *v*_1/5_ sheets to connect seamlessly and form new phases. The new phases have an atomically smooth phase boundary without out-of-plane distortion [129]. This strategy of constructing new phases offers an effective approach for the design and fabrication of new borophene-based materials with tunable properties, facilitating the realization of borophene applications. Two symmetric tilt GBs have been investigated in perfect borophene structures, indicating the domination of energetically favorable GB structures with *θ* = 60° (Figure 16a) [120].

## 4. Mechanical Behaviors and Properties of 2D Materials

### 4.1. Modulus and Strength

#### 4.1.1. Experimental Measurements via Nanoindentation in Atomic Force Microscopy

**Figure 17:**
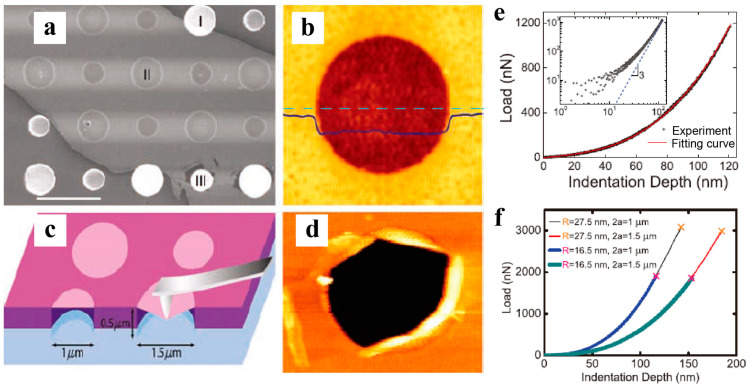
Experimental measurements of the moduli and strengths of 2D materials and associated curves were reproduced from Ref. [8]. (**a**) SEM image of graphene suspended on substrates with patterned circular wells. (**b**) Noncontact mode atomic force microscopy (AFM) image of suspended graphene. (**c**) Schematic illustration of AFM nanoindentation. (**d**) AFM image of fractured graphene. (**e**) Representative loading/unloading curves of nanoindentation. The fitting curve is based on an equation of $F=\sigma_{0}\pi \delta +Eq^{3}\delta^{3}/a^{2}$, where *F* is the indentation load, *δ* is the indentation depth, *σ*_0_ is the pretension, *E* is the modulus of indented membrane, *a* is the membrane diameter, and *q* is a dimensionless constant dependent on the Poisson’s ratio of membrane. (**f**) Typical results of AFM nanoindentation.

The in-plane tension of 2D materials is characterized by two important mechanical properties: elastic modulus and strength. Direct measurement of the mechanical properties of graphene was first performed using atomic force microscopy (AFM) nanoindentation by Lee et al. [8]. As shown in Figure 17, the graphene monolayers suspended over circular holes on substrates are indented by an AFM tip. A nonlinear stress–strain response for the graphene monolayer was obtained as *σ* = *Eε* + *Dε*^2^ through the indentation test. The Young’s modulus *E* and third-order modulus *D* were determined to be 1.0 ± 0.1 TPa and −2.0 ± 0.4 TPa, respectively. The intrinsic strength of pristine single crystalline graphene was 130 ± 10 GPa. This technique inspired similar characterizations and measurements of various 2D materials. Using this approach, researchers have measured the Young’s modulus and strength of h-BN, MoS_2_ monolayers, and few-layer phosphorene strips [130,131,132,133,134]. Few-layer phosphorene strips are observed to exhibit anisotropic mechanical properties [135]. Table 1 summarizes the mechanical properties of various 2D materials.

Although AFM nanoindentation has been widely adopted to measure the elastic properties and strength of 2D materials, it induces highly localized stress and strain underneath the AFM tip, which to some extent affects the strength measurement. A nanodevice was recently developed to achieve uniform tensile stress and used for mechanical testing of MoSe_2_ and graphene nanoribbons [136]. The obtained Young’s modulus of MoSe_2_ is consistent with that from DFT calculations, while the measured strength varies within a large range. Such considerable variation is attributed to undetectable pre-existing cracks introduced during the synthesis process [137]. Currently, mechanical testing of fragile 2D materials remains a significant challenge.

#### 4.1.2. First-Principle Calculations and MD Simulations

Atomistic simulation is an increasingly powerful tool for investigating the mechanical properties of nanostructured materials [138]. As an ab initio method, DFT calculations have been widely used to predict the elastic modulus and theoretical strength of graphene [139]. The predictions from DFT calculations agree well with the experimental measurements. The mechanical properties of various 2D materials beyond graphene have also been estimated via DFT calculations [140,141,142,143,144,145,146,147]. The relevant data are summarized in Table 1. Although the classic molecular dynamics (MD) simulations are less accurate than DFT, the MD simulations are allowed to address larger length scales (of the order of approximately 100 nm in 3D) and longer time scales (of the order of approximately 1 ns) than DFT. The accuracy and reliability of MD simulations are mainly determined by the interatomic interaction potential. Currently, reactive empirical bond order (REBO) [148] and adaptive intermolecular reactive empirical bond order (AIREBO) [149] potentials are common and highly recognized potentials used for simulating the properties of graphene. The Morse potential has been modified and used to describe the long-range interaction of the hydrocarbons at the extreme pressure [150]. The Tersoff potential [151] has been used to investigate the flexural phonons and thermal transport in graphene [152]. Reactive force-field (ReaxFF) [153] has been recently developed and used to study the atomic structural evolution of progressively reduced graphene oxide [64]. The Tersoff and Stillinger–Weber potentials are usually adopted for simulations of h-BN [154] and MoS_2_ [155]. As an increasing number of 2D materials emerge, corresponding empirical potentials are required for simulations of these emerging 2D materials. Atomistic simulation results showed that nearly all 2D materials exhibit high in-plane stiffness and theoretical strength due to the presence of covalent bonds in the plane. At a small strain, the elastic constants of graphene, h-BN, and MoS_2_ are isotropic, as indicated by Table 1. Phosphorene and borophene exhibited highly anisotropic Young’s modulus and Poisson’s ratio. At large strains, all 2D materials exhibit nonlinear responses, and their behaviors are dependent on the orientation, which is reflected by atomistic simulations rather than nanoindentation experiments.

**Table 1:** Moduli, Poisson’s ratio, and strengths of various pristine 2D materials from experiments and simulations. (zz and ac are abbreviations of zigzag and armchair, respectively).

Materials	Modulus	Poisson’s Ratio	Strength	Method	Ref.
graphene	1.0 ± 0.1 TPa	-	130 ± 10 GPa	AFM	[8]
	1050 GPa	0.186	121 (zz) GPa	DFT	[139]
			110 (ac) GPa		
h-BN	223–503 N/m (2–5 layer)	-	8.8–15.7 N/m (2–5 layer)	AFM	[131]
	292.1 N/m	-	71.7 N/m	Molecular mechanics	[131]
	780 ± 20 (zz) GPa	-	102 (zz) GPa	DFT	[144]
	773 ± 40 (ac) GPa		88 (ac) GPa		
MoS_2_	197.9 ± 4.3 (zz) GPa	0.21 (in-plane) 0.27 (out-of-plane)	24.7 (zz) GPa	DFT	[146]
	200.3 ± 3.7 (ac) GPa		25.1 (ac) GPa		
	129/118 N/m	0.29/0.31	-	DFT	[133]
	270 ± 100 GPa	-	22 ± 4 GPa	AFM	[134]
	330 ± 70 GPa (5–25 layers)	-	-	AFM	[130]
phosphorene	166 (zz) GPa	0.62 (zz)	18 (zz) GPa	DFT	[142]
	44 (ac) GPa	0.17 (ac)	8 (ac) GPa		
	58.6 ± 11.7 (zz) GPa	-	4.79 ± 1.43 (zz) GPa	AFM	[135]
	27.2 ± 4.1 (ac) GPa (14~28 nm)		2.31 ± 0.71 (ac) GPa		
borophene	166 (zz) N/m	-	12.98 (zz) N/m	DFT	[145]
	389 (ac) N/m		20.26 (ac) N/m		
borophene	163 (zz) N/m	0 (zz) –0.23 (ac)	12.39 (zz) N/m	DFT	[140]
	399 (ac) N/m		21.09 (ac) N/m		
*v*_1/12_	161 (zz) N/m	0.08 (zz) 0.09 (ac)	-	DFT	[140]
	208 (ac) N/m		-		
*v*_1/9_	212 (zz) N/m	0.14 (zz) 0.14 (ac)	18.77 (zz) N/m	DFT	[140]
	212 (ac) N/m		14.38 (ac) N/m		
*v*_1/8_	222 (zz) N/m	0.18 (zz) 0.17 (ac)	16.87 (zz) N/m	DFT	[140]
	216 (ac) N/m		15.50 (ac) N/m		
*v*_1/6_	210 (zz) N/m	0.17 (zz) 0.15 (ac)	15.50 (zz) N/m	DFT	[140]
	189 (ac) N/m		16.61 (ac) N/m		
*v*_1/5_	208 (zz) N/m	0.12 (zz) 0.11 (ac)	-	DFT	[140]
	196 (ac) N/m		-		
g-Si	71.2 N/m	0.401	6.0 (zz) N/m	DFT	[147]
			6.3 (ac) N/m		
b-Si	63.8 N/m	0.325	5.9 (zz) N/m	DFT	[147]
			6.0 (ac) N/m		
hexagonal silica	130.5 (zz) N/m	~0.5	38.3 (zz) N/m	DFT	[143]
	136.3 (ac) N/m		35.3 (ac) N/m		
haeckelite silica	84.3 (out-of-plane a_2_) N/m	-	29.4 (out-of-plane a_2_) N/m	DFT	[143]
	114.8 (in-plane a_2_) N/m		27.6 (in-plane a_2_) N/m		

#### 4.1.3. Theoretical Modeling

To describe the elastic properties of 2D materials, a molecular mechanics model and molecular structural mechanics model have been developed as a bridge to link atomic structures with macroscopic properties. Aiming at the linear elastic properties (such as Young’s modulus and Poisson’s ratio), Chang and Gao first adopted an effective stick-spiral model and derived analytical expressions to describe the elastic moduli of graphene and carbon nanotubes [156]. Beyond graphene, the properties of α-graphyne, β-graphyne, and γ-graphyne with more complicated arrangements are further investigated by using the stick-spiral model [157,158,159]. The theoretical model confirmed that graphene and graphyne with six-fold rotational symmetry have isotropic in-plane properties. The analytical model can be further extended to heteroelement nanostructures, such as h-BN sheets and nanotubes [160]. The predicted Young’s modulus and Poisson’s ratio from the theoretical model agree well with those obtained from atomistic simulations. Recently, Xiao et al. used molecular mechanics model to predict the fracture of a graphene and carbon nanotube and to further study the influence of defects on their tensile failure [161]. In recent years, a molecular structural mechanics model has been proposed and developed to investigate the mechanical behaviors of carbon nanostructures [162,163,164,165,166]. In a molecular structural mechanics model, the carbon nanostructure is regarded as the space frame and the covalent bond between atoms are modeled as a loading-bearing beam. A molecular structural mechanics model has been recently used to investigate the elastic properties and bulking of graphene [166].

#### 4.1.4. Influence of Defects (Grain Boundary and Vacancy) on Strength and Modulus

Defects have a significant influence on the mechanical properties of 2D materials. Even the mechanical properties of 2D materials can be tuned by controlling the density or distribution of defects. As mentioned above, vacancies are one of the most common structural defects in 2D materials. Interestingly, a controlled density of vacancies introduced in graphene by irradiation can significantly increase the Young’s modulus up to 550 N/m [167]. This phenomenon is attributed to the suppression of long-range flexural modes of graphene induced by vacancies under the framework of thermodynamic theory. As the density of vacancies increases, the softening effect becomes dominant, resulting in a reduction in the elastic modulus. A similar phenomenon was also observed by previous experiments of graphene irradiated by oxygen plasma [168]. However, the unexceptionally increased moduli of graphene with vacancies have not yet been completely supported by theoretical predictions [169,170,171].

Single crystalline graphene is usually obtained from mechanical exfoliation and has a limited in-plane length scale. In recent years, the chemical vapor deposition (CVD) growth approach has been used as a main synthesis method for the large-scale production of graphene. However, the CVD-grown graphene involves some GBs. Understanding the GB effects on the mechanical properties is essential for the applications of graphene. Recent nanoindentation tests have confirmed that the Young’s modulus and strength of CVD-grown high-quality graphene are comparable to pristine graphene [172]. Notably, the mechanical properties of polycrystalline graphene are determined by the quality (i.e., density and distribution of GB) of the samples. In experiments, an appropriate transfer technique is very important for the measurement of CVD-grown graphene. If the approach is improper during transfer, the GBs in polycrystalline graphene might be weakened, leading to contradictory conclusions from experimental measurements [105,173]. MD simulations and theoretical analyses have revealed that with evenly spaced defects, the strength of GBs generally increases with an increase in the tilt angle [174,175]. Such strengthening is attributed to the interaction between disclination dipoles in the GB [174,175]. The same trend has also been confirmed by recent experiments of bicrystalline graphene [176].

#### 4.1.5. Grain Size Effect on the Strength of Polycrystalline Graphene

**Figure 18:**
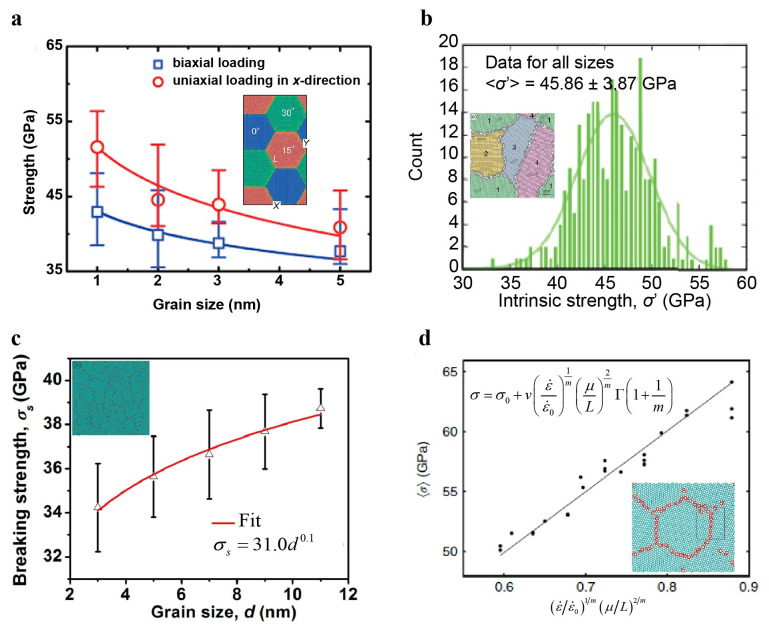
Influence of grain size on the strength of nanocrystalline graphene. (**a**) Grain size dependence of fracture strength. The inset shows an atomistic configuration of polycrystalline graphene. Reproduced from Ref. [177]. (**b**) Strength distribution of nanocrystalline grains with different grain sizes. The inset shows an atomistic configuration of nanocrystalline graphene. Reproduced from Ref. [179]. (**c**) Dependence of breaking strength on grain size. The inset shows the atomic configuration of nanocrystalline graphene. In (**c**), the simulation data are fitted as a power–law relationship between breaking strength *σ_s_* and mean grain size *d*. Reproduced from Ref. [180]. (**d**) Failure stress distributions of nanocrystalline grains with different grain sizes. The inset shows the atomic structure of polycrystalline graphene. In the equation inserted in (**d**), *σ* is the fracture strength, *σ*_0_ is a reference stress, *L* is the sample size, *μ* is the mean grain size, $\dot{\varepsilon }$ is the strain rate, ${\dot{\varepsilon }}_{0}$ is a reference strain rate, *m* is the Weibull modulus, *v* is a scale parameter and Γ( ) is the Gama function. Reproduced from Ref. [178].

The size effects of strength have been widely investigated in various nanostructured materials, for example, the classic Hall–Petch relationship in polycrystalline metals. Analogously, substantial attention has also been drawn to polycrystalline graphene [177,178,179,180]. As shown in Figure 18, the conclusions from different studies [177,178,179,180] about the grain size effects of nanocrystalline graphene strength seem to be contradictory. Song et al. constructed nanocrystalline graphene with hexagonal grains and tilt GBs involving pentagons, hexagons, and heptagons, performed MD simulations on these constructed samples, and finally obtained an analogous Hall–Petch relation between the strength of the nanocrystalline sample and grain size (in the range from 1 nm to 5 nm) [177]. The triple GB junctions in the sample with finite length GBs weaken the pristine structure [177]. The larger the grains are, the more severe stress concentrations are present at the triple GB junctions, resulting in significantly weakened strength [177]. Kotakoski and Meyer constructed polycrystalline structures with wavy GBs and random grain orientation and then ran equilibration for the constructed sample by annealing and quenching [179]. The nanocrystalline graphene after equilibration is very similar to the experimental sample. They also conducted MD simulations for the uniaxial tension of nanocrystalline samples with mean grain sizes of 3–16 nm. During simulations, cracks usually initiate at triple junctions. As a result, the strength of these nanocrystalline samples has no significant dependence on grain size but exhibits an apparent statistical distribution [179]. Using the Voronoi construction, Sha et al. generated nanocrystalline samples with larger in-plane sizes. Their simulations further confirmed that the GB junctions preferentially initiate cracks and are thought to be the main determinant of the strength of polycrystalline graphene [180] but show an inverse pseudo Hall–Petch relation [180]. Shekhawat and Ritchie constructed a considerable amount of nanocrystalline graphene for MD simulations and found significant statistical fluctuations in the toughness and strength of nanocrystalline graphene. They proposed a statistical theory developed based on the weakest-link model to understand the statistical variation in strength and toughness of nanocrystalline graphene [178].

The MD simulations mentioned above have revealed that nanocrystalline failure during tension typically initiates at GB junctions. The grain size effect of the strength largely depends on the detailed microstructures of the GBs. To date, nearly all present studies on the mechanical properties of polycrystalline materials are based on MD simulations, but physically realistic GB arrangements might have a certain discrepancy with the constructed samples used in modeling. Therefore, real experiments are still needed to investigate the relationship between polycrystalline graphene strength and grain size. Theoretical models of realistic GB structures are also required to further study the mechanical properties and behaviors of polycrystalline graphene.

### 4.2. Fracture Behaviors

#### 4.2.1. Model I Fracture of 2D Materials

The theoretical strength describes the maximum stress that can be sustained by perfect materials, while the fracture toughness is a measure of the material’s resistance to crack prorogation. Since crack-like flaws are inevitably introduced to 2D materials during synthesis or transfer, the fracture toughness of 2D is essential for practical applications of 2D materials [181]. Recently, MD simulations have revealed a brittle fracture in precracked pristine graphene with an energy release rate of 11.8 J/m^2^ [182], which is in good agreement with the results from coupled quantum/molecular mechanical modeling [183,184]. The classic Griffith theory is demonstrated to be applicable for the brittle fracture of graphene [185]. The crack orientation has a significant influence on the fracture loading. MD simulations showed that zigzag graphene exhibits lower fracture stress than armchair graphene [182,186]. MD simulations have also revealed that cracks prefer to propagate along the armchair or zigzag directions regardless of the crack orientation, which agrees well with experimental observations [187]. Such a preferred crack propagation direction is ascribed to the nonmonotonic dependence of graphene edge energy on the orientation [187].

**Figure 19:**
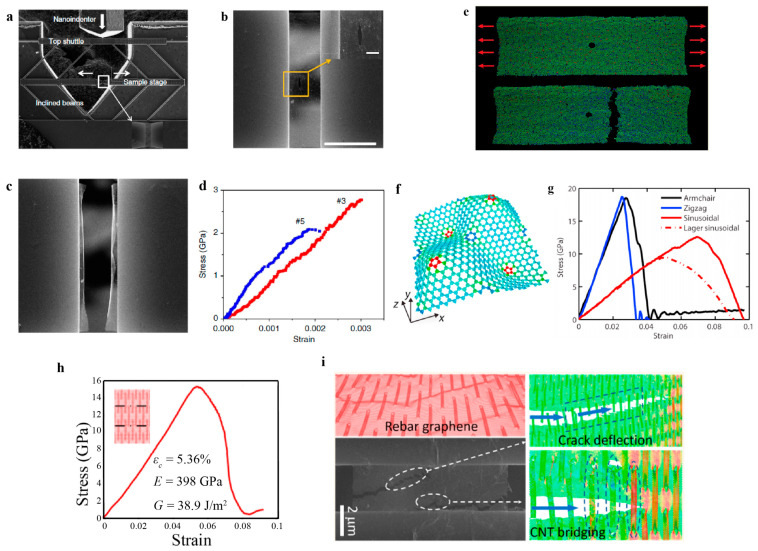
Fracture behaviors of various 2D materials. (**a**) SEM image of the nanomechanical device for the in situ fracture test. SEM image of monolayer graphene (**b**) before and (**c**) after a fracture. (**d**) Typical stress–strain curves of monolayer graphene. (**a**–**d**) reproduced from Ref. [182]. (**e**) Fracture behaviors of nanocrystalline graphene strips with a center hole, reproduced from Ref. [189]. (**f**) Atomic structure of sinusoidal graphene. (**g**) Stress–strain curves of sinusoidal graphene strips with an edge crack. (**f**,**g**) Reproduced from Ref. [194]. (**h**) Stress–strain curve of rebar graphene nanostrip with an edge crack from molecular dynamics (MD) simulation. (**i**) Fracture behaviors of rebar graphene obtained from MD simulations and experiments. (**h**,**i**) Reproduced from Ref. [206].

The fracture behaviors and properties of polycrystalline graphene have been recently investigated via in situ experiments and atomistic simulations [178,182,188]. Figure 19a–d show the in situ fracture test of 2D materials using nanomechanical devices. The samples are CVD-synthesized polycrystalline graphene containing a pre-crack introduced by focused iron beam (FIB) cutting. Under uniaxial tension, graphene exhibits brittle fracture initiating from a pre-existing flaw, leaving two flat edges. According to the Griffith theory, the stress intensity factor and energy release rate of polycrystalline graphene are estimated to be 4.0 MPa$\sqrt{m}$ and 15.9 J/m^2^, respectively [182]. Both intergranular and intragranular fractures are captured by the corresponding MD simulations [182]. Compared with pristine graphene, polycrystalline graphene has a higher release rate [178,182,188]. The reason is that the GBs in 2D materials can reduce the stress concentration near the crack tip and induce a wavy crack propagation path as well as crack branching. The toughness of polycrystalline graphene has a large fluctuation as the grain size varies, related to the detailed GB structures [178,188]. As shown in Figure 19e, when polycrystalline graphene nanostrips with an average grain size of 2 nm are subjected to tension, their fracture behavior becomes insensitive to a pre-introduced flaw [189]. Such insensitivity is further confirmed by the theoretical model based on the classic fracture mechanics theory, where there is no stress concentration near the flaw below a critical width, and the failure stress of the strip reaches up to the theoretical strength of the materials [190]. The critical width for flaw insensitivity is determined by the fracture energy, theoretical strength and Young’s modulus of the materials.

With the bursting of various 2D materials, their fracture behaviors have drawn increasing attention. MD simulations of the fracture of perfect MoS_2_ have shown that MoS_2_ has a much lower critical stress intensity factor than graphene [191]. Similar to graphene, the fracture toughness of perfect MoS_2_ is dependent on the orientation. Furthermore, the crack edge chirality is found to determine the crack propagation path because of the more complex atomic arrangement of MoS_2_. The zigzag crack prefers a straight path, while the armchair crack extends via a kink, leaving a zigzag edge [191]. For phosphorene monolayers, the energy release rate is comparable to that of graphene, as indicated by MD simulations [192]. Due to its highly anisotropic structure, the failure of phosphorene is attributed to the breakage of interlayer and intralayer bonds for armchair and zigzag loading, respectively [192]. The same experimental approach used for the fracture test of graphene is applied to monolayer MoSe_2_. However, MoSe_2_ is too brittle to sustain precracks through FIB cutting, resulting in a catastrophic failure during the cutting process. The lower surface energy of MoSe_2_ further suggests that MoSe_2_ is a more brittle material than graphene [137].

#### 4.2.2. Toughening Mechanisms

It is a long-standing challenge in the field of material science to achieve a material with both high strength and high fracture toughness. As a representative 2D material, although pristine graphene has very high stiffness and strength, it possesses a rather low fracture toughness. As crack-like flaws are inevitable in graphene, an effective toughening strategy is required to improve graphene’s toughness and ensure its safety and reliability during applications. The introduction of controlled topological defects has been proven to be an effective solution to toughness enhancement. It has been demonstrated that topological defects (such as disclinations and dislocations) give rise to apparent out-of-plane wrinkling, which can be utilized to generate 3D graphene structures and further alter the fracture properties [193]. Using the phase-field crystal method, a sinusoidal graphene structure is constructed by using patterned pentagons and heptagons. Such sinusoidal graphene with controlled topological defects is termed graphene ruga and has approximately twice the fracture toughness of pristine graphene (Figure 19f,g). Such toughness enhancement is achieved by crack shielding, crack bridging, and local curvature contribution [194]. Except for disclinations (i.e., isolated pentagons and heptagons), dislocations are also used for tailoring the fracture properties of 2D materials. MD simulations have shown that the dislocation has a significant shielding effect on the crack tip, resulting in graphene’s toughness enhancement. The crack–dislocation interaction is quantitatively described by continuum fracture mechanics theory and depends on the separation between the crack tip and dislocation [195]. Polycrystalline graphene exhibits a higher fracture toughness than defect-free graphene, ascribed to the interplay between GBs and cracks [178,182,188]. However, the introduced topological defects to some extent sacrifice the stiffness and strength of 2D materials. Overcoming the tradeoff between strength and toughness still requires a fundamental understanding of the toughening mechanism [196].

Another promising toughening method utilizes the interaction between nanocracks, i.e., establishing the concept of kirigami design. Recent MD simulations showed that patterning graphene with cuts results in a significant increase in yield and fracture strains [197,198]. The enhancement of ductility is also accomplished in the monolayer MoS_2_ kirigami structure [199]. As a recently emerging method applied in material science, machine learning is expected to design kirigami structures with targeted mechanical properties [200,201,202]. Optimal stretchable graphene structures have been designed and investigated via machine learning [203]. Recently, patterned graphene has been created by optical lithography and achieved large strains without failure [204]. Although multiple microcracks have a specific shielding effect in bulk materials [205], kirigami design at the nanoscale remains mysterious as to how it enhances the fracture toughness of 2D materials.

Nanocomposites are also a significant toughening strategy for 2D materials. One-dimensional carbon nanotubes (CNTs) are embedded in graphene to construct a hybrid structure, figuratively called “rebar-graphene.” Rebar graphene is demonstrated to remarkably enhance the fracture toughness of pristine graphene (Figure 19h,i). Such enhancement originates from the crack deflection and the bridging of the embedded CNTs captured by both experiments and simulations [206]. This novel mixed-dimensional composite provides new opportunities for 2D materials with targeted mechanical properties.

#### 4.2.3. Other Fracture Modes

The 2D materials exhibit other fracture modes (such as shear and tear failure) except mode I fracture. Due to the single-atom-layer structure, graphene under shear exhibits out-of-plane wrinkles, further affecting the in-plane mechanical properties. MD simulations have shown a reduction in fracture strength due to shear-induced ripples [207]. The formation and evolution of ripples have been revealed to be mostly dependent on the sample size but insensitive to temperature [208]. Under pure shear, graphene with one-dimensional Stone–Wales defects exhibits defect-guided wrinkling, which can tune the mechanical behaviors [209]. Similar to graphene, MoS_2_ also suffers out-of-plane ripples when subjected to shear loading. In particular, under the mixed mode of modes I and II, buckling cracks are generated due to out-of-plane deformation [191]. The propagation of buckling cracks is dependent on the phase angle for the mixed-mode [191].

Tearing is also a significant fracture mode that occurs during the fabrication and applications of 2D materials [210]. Moura and Marder investigated the tearing force to crack propagation in clamped and freestanding graphene monolayers via both numerical simulations and theoretical models [211]. The initial crack length and sample width are two important factors determining the tearing force [211]. Tight-binding MD simulations have revealed that the crack path is independent of the crack orientation and prefers to be along the armchair edge. However, for clamped graphene, the orientation and the initial crack length have a certain influence on the fracture patterns [211,212]. As the graphene is torn from the substrate, the armchair edges are experimentally found to be dominant in the torn sections [213]. Based on the classic fracture mechanics theory, a theoretical analysis has been adopted to describe the dynamic crack propagation velocity for a model-III fracture of graphene [214]. The theoretical model predicted that the steady-state fracture is a function of loading stress and lateral dimension, which agrees well with MD simulation results [214]. The unique single-atom-layer structure of 2D materials results in the coupling of the fracture under shear or tear with wrinkling/rippling, so theoretical models of these relevant fracture modes remain an open question.

#### 4.2.4. Fatigue Failure

Recently, Cui et al. used AFM to investigate the fatigue behaviors of graphene and graphene oxide [215]. It was found that monolayer and few-layer graphene possesses a fatigue life of more than 10^9^ cycles at an average stress of 71 GPa and a stress range of 5.6 GPa [215]. These fatigue experiments also revealed that the fatigue failure in monolayer graphene is global and catastrophic without progress damages, while the graphene oxide exhibits a local and progressive fatigue damage mechanism due to the presence of functional groups [215]. Cui et al. also conducted the in-situ cyclic loading of graphene-loaded polymer and observed the fatigue propagation at the graphene-polymer interface, which can be described well by a modified Paris’ law [216]. The fatigue failure of the graphene-polymer interface involves both in-plane shear and out-of-plane tear mechanisms [216]. These studies provided fundamental insights into the dynamic reliability of graphene and the graphene–polymer interface, which facilitates the applications of graphene in flexible electronics, multifunctional coatings and graphene-reinforced nanocomposites.

### 4.3. Piezoelectricity and Flexoelectricity

**Figure 20:**
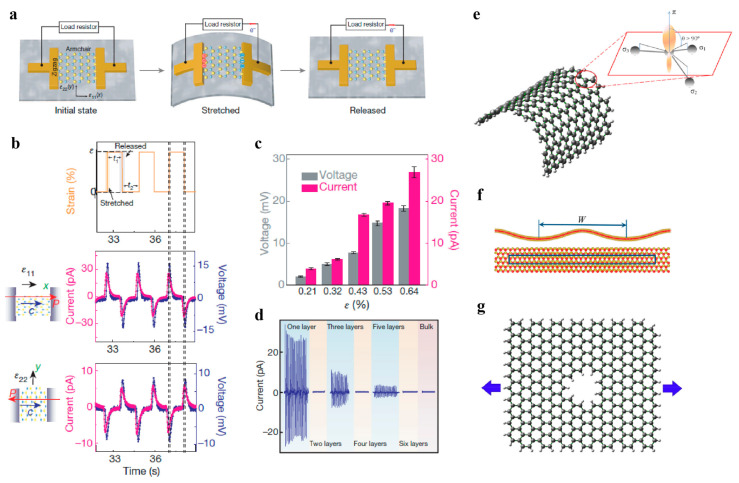
Piezoelectricity and flexoelectricity of 2D materials. (**a**) Schematic illustrations of the monolayer MoS_2_ piezoelectric device. (**b**) Piezoelectric current and voltage outputs for monolayer MoS_2_. (**c**) Piezoelectric responses of monolayer MoS_2_ at various applied strains. (**d**) Piezoelectric outputs for MoS_2_ with different atomic layers. (**a**–**d**) Reproduced from Ref. [218]. (**e**) Schematic illustration of out-of-plane polarization in curved graphene reproduced from Ref. [237]. (**f**) Schematic illustration of wrinkling-induced flexoelectricity, reproduced from Ref. [235]. (**g**) Schematic illustration of flexoelectricity introduced by nanopores, reproduced from Ref. [237].

Piezoelectricity can be exhibited among 2D crystals since the loss of inversion symmetry is caused by dimensionality reduction. With the rapid development of microminiaturized electromechanical systems, piezoelectric 2D materials have illuminated their potential applications in powering nanodevices, tunable optoelectronics, and sensors. For the MoS_2_ monolayer, oscillating piezoelectric outputs were detected under cyclic loadings (Figure 20a–c), which remained steady after numerous cycles [217,218,219]. Wu et al. confirmed a weakened piezoelectric effect with increasing layer number in multiple layers of MoS_2_ [205]. Piezoelectric responses only exist when the layer number is odd, as shown in Figure 20d. The corresponding piezoelectric coefficient of the MoS_2_ monolayer is measured to be *e*_11_ = 2.9 × 10^−10^ C/m by nanoindentation and a laterally applied electric field [220]. DFT calculations have been performed to predict the piezoelectric coefficients of various 2D materials, such as TMDCs and 2D oxides [221,222,223,224]. The relevant calculated data are summarized in Table 2, Table 3 and Table 4. These results provide versatile guidance for further investigations and applications on 2D materials for electronic and mechanical couplings. The predictions from DFT calculations have indicated that a larger ratio of chalcogen anion and metal cation polarizability leads to larger piezoelectric responses among TMDC monolayers [221,224].

2D structures with inversion symmetry result in the absence of the piezoelectric effect, limiting their potential applications in electronics. Fortunately, the large aspect ratio of 2D materials provides a possibility for chemical doping to induce piezoelectricity. The inversion symmetry of pristine graphene can be broken by saturating graphene with heterologous atoms on only one side, which has been experimentally demonstrated [225,226]. DFT calculations showed that the piezoelectric coefficients of the doped graphene structures are of the same magnitude as those of intrinsic piezoelectric 2D materials [227]. Patterned doping has been proven to be an effective approach for nanoscale controlling of electromechanical properties [227].

The abovementioned piezoelectricity arises from polarization caused by uniform strain, while flexoelectricity describes the coupling of electronic polarization and strain gradient. Flexoelectricity is exhibited in all crystalline materials regardless of the lattice symmetry. However, the detection and applications of the flexoelectric effect require a large strain gradient or flexoelectric coefficient. The flexoelectric effect is nearly negligible in bulk materials. As the sample scale decreases down to micrometers or nanometers, the resultant large strain gradient leads to a strong flexoelectric effect. In recent years, nanoscale flexoelectricity has therefore drawn considerable attention [228,229].

Note that for single or few atomic layer structures along the thickness direction, the flexoelectricity of 2D materials is induced from the in-plane and out-of-plane strain gradients, as illustrated in Figure 20e–g. Much attention has been drawn by introducing curvature in 2D structures, which has been predicted to create out-of-plane polarization (Figure 20e). DFT calculations showed that curved graphene exhibits dipole moments because of the redistribution of electrons [230,231]. It was further found that a decrease in the radius of curvature leads to stronger flexoelectricity in graphene, while the orientation shows less influence on polarization [231]. Such dependence of the flexoelectric effect on curvature in other 2D systems (including carbon nanocones [232] and h-BN [233,234]) has also been elucidated. The curvature in bilayer h-BN leads to a greater in-plane flexural polarization compared with the monolayer structure [234]. For various TMDCs, wrinkling and corrugation similarly cause polarization by a large strain gradient (Figure 20f), and the induced flexoelectricity is much stronger than that in graphene [235].

Another intriguing approach to create in-plane flexoelectricity is the introduction of noncentrosymmetric nanopores. Tight-binding modeling and DFT calculations have been adopted to investigate graphene monolayers containing triangular or trapezoidal pores (Figure 20g) [236,237]. Due to the existence of the nanopores, a strain gradient is generated in graphene even under uniform strain. The apparent polarizations are then induced due to the flexoelectric effect. The patterned defects endow the intrinsic nonpiezoelectric 2D materials with tunable piezoelectricity. In addition to graphene, a 2D structure named graphene nitride (g-C_3_N_4_) is considered an ideal candidate because of its natural presence of triangular nanopores [238]. Both DFT calculations and piezoresponse force microscopy (PFM) experiments have confirmed g-C_3_N_4_ to be piezoelectric. More importantly, such piezoelectricity does not vanish in structures with multilayers, which is convenient for its fabrication and applications [239].

**Table 2:** Piezoelectric coefficients of various 2D materials from DFT calculations or experiments [221].

Material	*e*_11_ (pC/m)	*d*_11_ (pm/V)	Material	*e*_11_ (pC/m)	*d*_11_ (pm/V)
2*H*-CrS_2_	543	6.15	2*H*-TaSe_2_	250	3.94
2*H*-CrSe_2_	575	8.25	2*H*-TaTe_2_	207	4.72
2*H*-CrTe_2_	654	13.45	BeO	132	1.39
2*H*-MoS_2_	362	3.65	MgO	230	6.63
	290(Exp) [220]	-	CaO	155	8.47
2*H*-MoSe_2_	383	4.55	ZnO	266	8.65
2*H*-MoTe_2_	467	7.39	CdO	333	21.7
2*H*-WS_2_	243	2.12	BN	139	0.61
2*H*-WSe_2_	257	2.64	BP	240	2.18
2*H*-WTe_2_	323	4.39	BAs	204	2.19
2*H*-NbS_2_	211	3.12	BSb	206	3.06
2*H*-NbSe_2_	222	3.87	AlN	223	2.75
2*H*-NbTe_2_	184	4.45	GaN	148	2
2*H*-TaS_2_	267	3.44	InN	224	5.5
GaS [209]	134	2.06	-	-	-
GaSe [209]	147	2.30	-	-	-
InSe [209]	57	1.46	-	-	-

**Table 3:** Piezoelectric coefficients of graphene doped by different atoms [227].

Atom(s)	*e*_31_ (pC/m)	*d*_31_ (pm/V)	Atom(s)	*e*_31_ (pC/m)	*d*_31_ (pm/V)
Li	55	0.15	F	−26	0.0018
K	52	0.23	H, F	−31	0.034
H	20	0.11	F, Li	30	0.3

**Table 4:** Piezoelectric coefficient for unstable and metastable structures [221].

Material	*e*_11_/*e*_31_	*d*_11_/*d*_31_	Material	*e*_11_/*e*_31_	*d*_11_/*d*_31_
PbO ^a^ (p)	280	73.1	GaAs ^b^	49/8.2	1.5/0.125
AlP ^a^ (p)	3.5	0.09	GaSb ^a^	33.2/0.8	1.42/0.016
AlAs ^a^	12.7/40.1	0.38/0.568	InP ^b^	0.5/25.1	0.02/0.390
AlSb ^a^	19.9/18.6	0.79/0.351	InAs ^b^	1.7/12.6	0.08/0.248
GaP ^b^	52.6/25.9	1.29/0.310	InSb ^a^	17.9/2.3	1.15/0.058

^a^ Unstable structure. ^b^ Metastable structure (within 10 meV/atom).

### 4.4. Friction and Interlayer Shear

#### 4.4.1. Sliding Friction on Surfaces

Bulk materials with laminate structures are widely used as solid lubricants because of interlayer weak bonding by van der Waals forces. The high relative surface area and good thermal properties of 2D materials make them ideal candidates for lubricants in nanoscale electronic or mechanical systems [240]. The AFM measurements showed that bilayer graphene lowers the friction by a factor of two compared with single-layer graphene. Such an unexpected phenomenon may be interpreted by the enhancement of electron–phonon interactions in single-layer graphene, leading to more efficient energy dissipation [241]. Four different 2D crystals also exhibited the same trend of decreasing friction with increasing sample thickness. Such a trend always maintains various electronic and vibrational properties of 2D materials and different experimental conditions such as humidity, tip–substrate interactions, scan speed, and sizes. Until now, there has been no fundamental mechanism to explain this trend. A mechanistic interpretation from the out-of-plane puckering effect may be reasonable for such friction behavior. When the tip slides on the surface, the 2D materials locally pucker and contact the tip because of their low bending stiffness and interactions with the tip, leading to higher friction. As the bending rigidity increases for the thickened sample, the puckering effect is less predominant. This weakened out-of-plane puckering reduces the tip–sheet contact area and further lowers the friction, which is consistent with the experimental observations and finite element modeling [242].

The friction measurements via frictional force microscopy (FFM) of the chemically modified graphite showed an unexpected increase with decreasing normal load, indicating a negative friction coefficient [243]. The corresponding MD and finite element simulation results have further revealed that the delamination of the surface layer of graphite is responsible for this effect [243]. The effective negative friction coefficient is intrinsically attributed to the competition between the tip–top layer of graphite interaction and the elastic energy associated with the deformation inside graphite [244].

#### 4.4.2. Interlayer Shear/Sliding

2D materials generally exist in the forms of single layers, few layers, or multiple layers. It is necessary to investigate the interlayer shearing and sliding of 2D materials to further explore their assembly and to ensure their performance and reliability during applications. When two graphite sheets are in incommensurate contact, they can easily slide with each other, exhibiting superlubricity due to the ultralow shear strength between two graphite sheets [245]. Such superlubricity has been observed between graphite flakes attached to AFM tips and graphite substrates [246]. Using scanning tunneling microscopy, the graphene nanoflakes in incommensurate states are found to slide freely on the graphene surface to reach commensurate positions, further confirming that the superlubricity is related to the incommensurability [247]. Recent MD simulations showed that unique friction behaviors (especially superlubricity) have an apparent dependency on atomic arrangements and orientations between graphene layers [248,249]. The support stiffness has been proven to be an effective factor to tune the superlubricity of interlayer sliding in graphene [249]. Most recently, pressurized microscale bubble loading tests on bilayer graphene have been used to measure the interlayer strength of 40 kPa between two graphene layers [250]. The ultralow interlayer strength of bilayer graphene originates from the incommensurable interface between two graphene layers [250].

#### 4.4.3. Friction Modulation

The defects of 2D materials alter the surface states of the structure, leading to apparent modifications of their friction behaviors. FFM measurements showed that charged iron irradiation-induced defects in single-layer graphene efficiently enhanced friction [251]. MD simulations have revealed that the presence of a single vacancy and Stone–Wales defects significantly affect the friction behaviors of graphene. For stacked few-layer graphene, the friction force of a structure with surface defects is higher than that with internal defects [252]. For sliding between bilayer graphene, the Stone–Wales defect increases the interlayer friction, while the vacancy presents the opposite along a certain sliding orientation for incommensurate stacking [248].

A wide range of applications of graphene results in complicated environmental exposure. When exposed to humid conditions, graphene was observed to present increased friction compared with that in a dry atmosphere, which becomes more apparent with aging. Experiments and simulations have indicated that increasing friction is associated with variations in surface energy [253]. Furthermore, sliding friction in complete liquid environments has also been investigated as one of the possible application situations. FFM measurements and MD simulations have revealed that the friction behaviors of graphene persist almost the same in water or ultrahigh vacuum environments [254]. In the water environment, the water molecules stochastically affect the friction behaviors of graphene due to the breakage of hydration layers at the interface, while they facilitate the atomic resolution of the lattice [254].

When 2D materials are attached to substrates, the interaction strength between these systems has proven to be a determinant of their friction behaviors [229]. Modification of substrates has been proposed as an efficient and universal method to control and tune friction. A novel approach to adjust the friction behaviors of 2D materials is plasma treatment towards substrates [255]. The plasma treatment strengthens the adhesion of the interface by introducing abundant functional groups into the substrate, leading to a decrease in the friction coefficient of graphene on the substrate. The decreased friction is attributed to the suppression of the out-of-plane deformation by strong interface adhesion between graphene and the substrate, while the surface roughness plays a minor role [255]. To further clarify the surface roughness effects, the friction behaviors of graphene sliding on nanoparticle films have been investigated experimentally [256]. The weak interaction between graphene and the substrate causes a partial suspension of graphene on the substrate with nanoscale roughness. Except for the adhesion properties between graphene and the substrate, the curvature of the AFM tip and the contact area of the interface have a significant influence on the friction behaviors of graphene [256]. An atomically flat substrate has been found to pronouncedly reduce the surface roughness of 2D materials deposited on the substrate, resulting in a remarkable enhancement in lubricity [257].

### 4.5. Van der Waals Interaction between 2D Materials and Substrate

The booming study of 2D materials has attracted much attention beyond the simple structures and properties of graphene and its analogs. Indeed, the interactions between 2D materials and substrates and between heterogeneous multilayers have attracted enormous interest due to their distinct properties and exciting applications.

#### 4.5.1. Tunable Band Gap of 2D Materials on a Substrate

Strain engineering of 2D materials has brought a new dawn of fundamental mechanisms and thrilling applications. Graphene, although it exhibits extraordinary electronic properties, lacks a band gap, making it intricate in practical applications. Various approaches have been proposed to modify the band gap in graphene to broaden its applications, among which the mechanical strain method has drawn much attention. First-principle calculations have indicated that the opening of the band gap is expected through asymmetrical strain. When uniaxial tension is applied to graphene, a tunable band gap of graphene can be achieved, but the symmetrical biaxial strain has a negligible influence on the band gap opening [258,259]. The original existence of a band gap in TMDCs endows them with more appropriate materials in electronics and optoelectronics than graphene. This direct band gap can be tuned on demand via strain engineering. Ab initio simulations showed that unlike graphene, TMDCs exhibit a remarkable decrease in band gap when subjected to both tensile and shear strains [260,261,262]. Semiconductor-to-metal transitions can then be achieved at different strain states for various 2D structures [260,261,262].

Due to the atomic nature of 2D materials, precise strain control over monolayer or few-layer structures remains challenging. Currently, the strain engineering of 2D materials can be achieved by epitaxial growth, thermal strain, and flexible substrates [263]. The epitaxial growth and thermal strain originate from the lattice mismatch and the thermal expansion mismatch in CVD-grown 2D materials, which has a certain limitation on the strain magnitude for specific materials. Among these approaches, transferring 2D materials to flexible substrates provides a more straightforward method for controlling mechanical strain. The required strain is easily realized and measured for the attached 2D materials by stretching the underlying substrate. The perturbation of mechanical strain on the band structure of 2D materials can then be examined experimentally [258,264]. Through Raman spectroscopy, the electronic transformations of monolayer graphene and MoS_2_ under uniaxial strain have been indirectly observed [258,264]. Therefore, strain engineering has been demonstrated as an efficient tool for tuning the electronic properties of 2D materials.

#### 4.5.2. Van der Waals Interface of Heterostructures

The evolution of research on graphene and isolated 2D crystals has shifted focus to atomic layers vertically stacked by different 2D materials. Van der Waals-bonded heterostructures are emerging with unique structural and electronic varieties, resulting in their possible applications in novel electronic and photoelectronic devices [265,266]. Silicon and its oxide are common substrate candidates as supports for graphene due to their versatility and accessibility. However, SiO_2_-supported graphene presents pronounced weakened carrier mobility, which results from the surface roughness and interlayer contamination. Graphene laid on a single crystalline h-BN has demonstrated a remarkable enhancement in carrier mobility compared with that on SiO_2_ substrates [267]. This success has stimulated the development of heterostructures with novel functionalities. The h-BN monolayer can be adopted as gate dielectrics to achieve a metal-insulator transition in graphene sandwiched by h-BN [268]. The heterostructures constructed by inserting a monolayer TMDC layer between two graphene layers exhibited improved photoabsorption and photocurrent response, enabling a more efficient photovoltaic device compared with isolated TMDC crystals [269]. The combination of graphene with insulating 2D crystals has been thought to be an available approach for nanoscale tunneling transistors. The high on-off ratios for the field-effect transistors achieved by h-BN and MoS_2_ sandwiched with graphene may enlighten further development [270].

The tunable electronic and optoelectronic properties of heterostructures can be achieved through the crystallographic orientation between stacking crystals. For the graphene on the h-BN substrate, the similar hexagonal structure with a 1.8% difference in lattice constant results in a moiré structure when joining. The induced periodic potential forms a secondary Dirac point [271]. Rotation-dependent structure and electronic property modulations have recently been confirmed in stacked 2D monolayers with analogous atomic arrangements, such as MoS_2_/MoSe_2_ [272] and MoS_2_/WSe_2_ [273]. However, the contamination absorbed between interlayers during the assembly process may degrade the intrinsic properties of heterostructures. Fortunately, various adsorbates at the interface of graphene with h-BN and TMDCs are segregated in bubbles due to van der Waals interactions, leaving an atomically sharp interface [274,275]. Despite atomic flatness, graphene stacked with laminated oxides lacks the self-cleaning phenomenon due to their weak affinities [274,275].

## 5. Conclusions and Perspectives

In this review, we summarize recent advances in experimental, computational, and theoretical studies on the mechanical behaviors and properties of various 2D materials, including their tension and fracture behaviors, piezoelectricity, flexoelectricity, friction, interlayer shear properties, and van der Waals interactions. We further emphasize that some common defects in 2D materials have a profound influence on their mechanical behaviors, suggesting tunable properties by defect engineering. Although graphene discovery has stimulated prosperous studies of 2D materials, there are still open questions calling for further exploration.

Numerous experimental characterizations and mechanical tests have been mainly performed on graphene, MoS_2_, or h-BN monolayers, while experiments have seldom been conducted for other newly synthesized 2D crystals due to technological limitations and difficulties. The extraordinary properties of novel 2D crystals largely remain as theoretical proposals. In addition, the experimental approach is quite limited for mechanical measurements of 2D materials. The pioneering work of nanoindentation tests for graphene has become the most frequent mechanical characterization approach for 2D materials. However, nanoindentation induces local deformation and only reveals an average property for anisotropic 2D materials. A more effective analytical model is needed for extracting the intrinsic mechanical properties of 2D materials [276,277]. For uniaxial in-plane loading for uniform tension or fracture tests, an elaborate nanodevice and proper transfer techniques are required because these techniques are critical for measuring 2D materials.

The defect engineering or topological design of 2D materials has drawn much attention for tunable mechanical or electronic properties, but there is a general lack of fundamental understanding of intrinsic mechanisms for defective structures. For the mechanical strength, the disagreement of various defects’ influences calls for experimental verifications and further theoretical explanations. The toughening of brittle 2D crystals has been realized via topological design. It is then necessary to further probe the optimization of fracture properties by controlling patterned defects, which may be a heavy burden due to variant design parameters. Further explorations of tailoring fracture behaviors rely mostly on understanding toughening mechanisms and interactions between cracks and defects, which until now remain mysterious. Although the introduction of patterned defects into pristine 2D crystals has been given by previous studies [278,279], the gap between the targeted length scale of current experiments and numerical simulations is still too large to fulfill. Therefore, the verifications of theoretical modeling become challenging.

Investigations on 2D materials have extended from specific performances to taking them as platforms for heterostructure studies. In addition, unstable 2D structures require encapsulation by other 2D materials with chemical inertness for practical applications. The typical assembly process of heterostructures contains the layer-by-layer transfer of monolayer 2D crystals. This procedure has high inefficiency, which urges the further development of assembly techniques. The effort of epitaxial growth on other 2D materials as templates endows the possibility of large-scale synthesis for industrial applications [280,281]. Recent studies on heterostructures composed of bilayer graphene have revealed that a magic twisting angle between two layers leads to extraordinary electronic properties such as superconductivity [282,283]. The unconventional fascinations are attributed to complex interactions between vertically stacked layers realized by accurate control of the twisting angle. To realize the ambition of heterostructures in electronics and optics, a thorough understanding of mechanical features is essential. However, the van der Waals interaction effect on the mechanical properties and the fracture and stress transfer mechanism still lack investigation.

A new era of nanotechnology has been seen since the first discovery of graphene monolayers. After extensive efforts in the scientific community, 2D materials have been revealed as marvelous candidates for many applications, from conventional mechanics, electronics, and optics to novel biomedicine, drug delivery, and energy storage. The emergence of vertically stacking heterostructures has broadened the studies of 2D materials beyond monolayer crystals to multilayer structures by human design. An expanding number of family members and defect engineering methods further provide multiple choices for tailoring the mechanical, physical, and chemical properties of 2D material systems, which can be used for future practical applications.

## Data Availability

Data sharing is not applicable to this article.
